# Retraction for: Exosomes derived from human umbilical cord mesenchymal stem cells promote osteogenesis through the AKT signaling pathway in postmenopausal osteoporosis

**DOI:** 10.18632/aging.205437

**Published:** 2023-12-14

**Authors:** Shi-Wei Ren, Guang-Qing Cao, Qing-Run Zhu, Min-Gang He, Fang Wu, Su-Mei Kong, Zhao-Yan Zhang, Qiang Wang, Feng Wang

**Affiliations:** 1 Department of Orthopedics, The Provincial Hospital Affiliated to Shandong First Medical University, Jinan 250014, Shandong, China; 2 Department of Spine Surgery, The Second Hospital, Cheeloo College of Medicine, Shandong University, Jinan 250033, Shandong, China; 3 Department of Gastrointestinal Surgery, Shandong Tumor Hospital and Institute, Shandong First Medical University and Shandong Academy of Medical Sciences, Jinan 250117, Shandong, China; 4 Department of Health, 960th Hospital of PLA, Jinan 250031, Shandong, China

**Keywords:** postmenopausal osteoporosis, exosomes, human umbilical cord mesenchymal stem cells, osteogenesis

**This article has been retracted:** Aging has completed its investigation of this paper. We found that the data shown in **Figure 4** are duplications of previously published data from papers by Kuang et al 2021 [1] and Lv et al 2020 [2]. The proliferation experiment images for the PMO and PMO+Exo groups in **Figure 4A** were duplications of images from different experiments in [1]. The PMO+Exo panel in **Figure 4E,** presenting data from ALP assays to evaluate osteogenesis in different treatment groups, contains duplicated images of ALP staining of different cell types and treatment from [2]. The authors found that the data were wrongly copied and misused in this paper, as Dr. Ren worked in the same laboratory as Dr. Kuang and had access to his data. The corresponding authors have contacted and apologized to Professor Lv and Dr. Kuang for the plagiarism of their published data and inconvenience caused by their slack supervision. All the authors agreed to retract this paper.
